# Alliance matters: but how much? A systematic review on therapeutic alliance and outcome in patients with anorexia nervosa and bulimia nervosa

**DOI:** 10.1007/s40519-021-01281-7

**Published:** 2021-08-10

**Authors:** Janina Werz, Ulrich Voderholzer, Brunna Tuschen-Caffier

**Affiliations:** 1 Department of Clinical Psychology and Psychotherapy, Albert-Ludwigs-University, Freiburg, Germany; 2 Schön Klinik Roseneck, Am Roseneck 6, 83209 Prien am Chiemsee, Germany; 3 Department of Psychiatry and Psychotherapy, University Hospital, Ludwig Maximilians University, Munich, Germany; 4 Department of Psychiatry and Psychotherapy, University Hospital, Albert-Ludwigs-University, Freiburg, Germany

**Keywords:** Therapeutic alliance, Working alliance, Anorexia nervosa, Bulimia nervosa, Eating disorders, Systematic review, Outcome

## Abstract

**Purpose:**

Patients with eating disorders (ED) pose a high-risk group regarding relapse. The understanding of factors contributing to a better outcome is much-needed. Therapeutic alliance (TA) is one important, pantheoretical variable in the treatment process, which has shown to be connected with outcome. This review looks into a possible predictive effect of TA on outcome as well as related variables.

**Methods:**

A systematic review with pre-determined inclusion criteria following the PRISMA guidelines was conducted for studies published since 2014. Three previous reviews including studies up until 2014 were analyzed for studies matching our inclusion criteria. A total of 26 studies were included.

**Results:**

The results were heterogeneous between different patient groups. Regarding the predictive effect of TA, in adolescent samples, the TA of either the patients or their parents seems to impact outcome as well as completion. For adults, results are mixed, with a tendency to a greater impact of TA for anorexia nervosa (AN) patients, while some samples of adult bulimia nervosa (BN) patients did not find any relation between TA and outcome.

**Conclusion:**

The effect of TA on clinical outcome depends on the patient group. TA has a greater impact on adolescents, irrespective of diagnosis, and on adults with AN. The examined studies have different limitations which include small sample sizes and questionable study design. The examination of motivation as a potential influencing factor is recommended.

**Level of evidence:**

Level I, systematic review.

## Background

Eating disorders (ED) are complex disorders affecting the emotional, cognitive, and physical well-being of those affected. Looking into large international epidemiological studies, between 0.9% and 4% of women suffer from anorexia nervosa (AN) at some point during their lifetime, and 0.5–2% are affected by bulimia nervosa (BN) [1–3]. AN and BN affect the life of patients in the most serious of ways and the success of treatment is often far from satisfactory. For AN, remission rates after disorder-specific psychotherapy vary between studies, ranging from 30 to 50%, with around 30% of patients experiencing a chronic course of disease [4, 5]. The standardized mortality ratio is around 5.5 [6–8]. For BN, the remission rates seem to be similar with around 40% remission after cognitive behavioral therapy [9]. While the mortality rate is lower than in AN, with a standardized mortality ratio between 1.5 and 2, the effects of the disorder are still quite severe [6–8]. The risk of a cardiovascular disease is ten times higher than for a control group without BN in the 12 years following an admission for BN [10]. Hence, the further improvement of treatment success is an important objective to achieve better perspectives for affected patients. There are several equally effective psychotherapeutic approaches for AN and BN (for an overview, see [11–15]). It seems that there is a lesser need for new approaches but rather for a refining of existing psychotherapy to enhance its efficiency. International guidelines stress the importance of process-related variables as important factors influencing the outcome of treatment [16, 17].

Orlinsky and colleagues [18] did extensive research in this area, culminating in the “Generic Model of Psychotherapy”, which includes various process variables classified in different groups. In their recent systematic review of those variables regarding the treatment of eating disorders, Brauhardt and colleagues [19] came to the conclusion that research focusing on process-outcome associations for the treatment of ED is limited, with the influence of some variables still unclear. They state the necessity of future research looking closer into the often complex and bidirectional relationship between process and outcome variables. One of those variables emphasized by Brauhardt and colleagues as well as international guidelines [16, 17, 19] is the therapeutic alliance (TA). TA was found to be a reliable predictor of outcome for various disorders in some large meta-analysis, positively influencing outcome even after controlling for possible confounding variables like patient intake characteristics and other treatment process variables [20, 21]. However, the association appears to be smaller for patients with ED than with other disorders like depression or anxiety [20–22]. Brauhardt and colleagues [19] therefore argue that it is important to consider differential effects of TA for different subgroups of ED patients.

As TA describes a latent, not directly observable concept, it needs some kind of conceptualization. While the concept of a special bond between therapist and patient reaches far back to the beginning of psychotherapy [23], Bordin [24] was the first researcher who outlined a pantheoretical conceptualization of the therapeutic alliance, which is still the basis of our “modern” understanding of TA. In his view, TA is a precondition for treatment—necessary (though not sufficient) to reach a good treatment outcome. His model consists of three elements: agreement on the goals of treatment, agreement on the tasks necessary to be accomplished and the quality of the bond between patient and therapist. With this conceptualization, TA was operationalized in a way that enabled the development of tools to measure the quality of TA quantitatively. In the following years, various instruments for the assessment of TA were developed, based on slightly different approaches to TA. Luborsky and colleagues were the first group that designed such a tool, the “Helping alliance questionnaire” (HAQ; in some studies, it is referred to as the “Helping relations questionnaire/HRQ”) [25]. In their first 11-item version of the HAQ, some questions concerned the previous benefit from the therapy, which was criticized to be a confounding variable. Therefore, in their revision and development of the HAQ-II [26], they excluded those items and adapted the questionnaire to fit more into Bordin’s model of TA. Other commonly used assessment tools for TA include the California Psychotherapy Alliance Scales (CALPAS) [27] and the Vanderbilt Therapeutic Alliance Scale (VTAS) [28]. Another questionnaire based on Bordin’s theory is the “Working alliance questionnaire” (WAI) [29]. The WAI is a short and widely used measure that exists in different versions: for the patient’s view, the therapist’s view, and as an observer rating. It consists of three scales that correspond with Bordin’s model one-to-one: “tasks”, “goals”, and “bond”. For each of those elements, patients with ED may pose specific challenges in building a strong and reliable TA.

Reaching an agreement on the domain “goals” may be especially difficult with AN patients, as a high ambivalence and pronounced ego-syntonicity are part of the pathology [30]. Conflicts may arise between therapist and patients concerning, for example, the targeted weight or the composition of food. But even with agreed on goals (“not having an eating disorder anymore”), sometimes the tasks necessary to fulfill these goals may lead to problems, as the link between needing to restore weight and increasing calorie intake/reducing exercise is often hard to accept for AN patients. Similarly, BN patients also may pose a challenging group for establishing agreement on tasks, as their fear of weight gain is a central part of the symptomatology [31], leading to ambivalence regarding the cessation of purging behavior. Hence, there seem to be some difficulties in building a strong therapeutic alliance, that lie in the very nature of AN and BN, posing a difficult and often energy-consuming problem for therapists.

Regarding the different patient groups that are comprised in overall EDs, we considered carefully which of these groups to include in our review. As we wanted to maintain clarity, comprehensibility, and comparability, we decided to only include patients suffering from AN and BN. This decision was also driven by the already quite heterogeneous findings reported in the previous reviews regarding TA and outcome in eating-disorder patients, even for AN and BN patients [19, 32–34]. Moreover, the specific challenges concerning TA for AN and BN patients described above lie in the high ambivalence regarding change and therefore probably differ for those with BED. While the task of changing symptomatic behavior may be as difficult as for other EDs, lower ego-syntonicity as well as higher insight into the burden of disease should increase the agreement on the goals and tasks of therapy and hence the TA. Additionally, studies suggest more differences between BED and other ED in some important aspects, for example affective dynamics [35] or interoception [36].

Most research focuses on TA in individual therapy, rather than group therapy [37]. As there are multiple relationships formed in a group setting, the concept of a dyadic TA is not applicable there, and more complex conceptualizations are needed. As the related, but distinct concept of group cohesion poses a confounding variable, a comparison between TA in individual and group settings cannot easily be made. Therefore, we chose to look only into studies conducted in an individual setting. However, we extended that by also including studies using a family-based concept. As family-based therapy (FBT) is a standard treatment for adolescents with EDs, the exclusion would lead to a big gap regarding adolescent patients. Moreover, while TA in therapeutic groups is better conceptualized as or at least confounded by group cohesion, the TA in FBT can be measured the same way as in individual therapies, with the only difference of parents being (additionally) asked to rate their TA with the therapist.

The question of whether a good therapeutic alliance is related with better outcome and less attrition in eating-disorder treatment is still unanswered. Previous systematic reviews [19, 32, 34] as well as one meta-analysis [33] have shown the heterogeneous results of the studies conducted so far, with only a few other variables examined for their influencing effect and large differences in samples, settings, and designs. In their meta-analysis Graves et al. [33] found indications of a bidirectional relationship of symptom change and TA over time, stating that there is more research necessary to understand this complex and time-dependent relationship. While the meta-analysis examined specific research questions, the prospective of previous systematic reviews mainly included a systematic but quite exploratory description of studies, without any specific research questions. Our goal was to enhance the impact of our systematic review by asking and answering clearly and previously defined research questions, in contrast to those previous systematic reviews. Thereby, we were aiming at a thorough analysis regarding the studies’ outcome to enable their comparison as well as specific conclusions regarding subsamples. However, as the current state of research is quite vague, with heterogeneous results even as to the basic question if there is any relationship between TA and outcome and hardly any findings on variables influencing such a relationship, we compiled broadly defined questions. Previous reviews as well as the meta-analysis only include studies up until 2014. As the research focus has moved more into process-outcome variables in recent years, this article aims to provide an update on all research available regarding the relationship between treatment outcome and therapeutic alliance in patients with AN and BN as well as insights concerning additional variables that may have an influencing effect. To achieve a comprehensive summary of all research published on this topic and build up on the previous research, we decided to also include the studies assessed in those reviews into our analysis.

The following research questions are being asked:

1. Are therapeutic alliance and treatment outcome related and does therapeutic alliance positively predict treatment outcome?
2. Which additional variables influence the relationship between therapeutic alliance and treatment outcome?
3. Are there differential effects of TA on outcome, dependent on the sample characteristics (specifically: age and diagnosis)?

## Methods

The selection process for studies included in this review was conducted using a two-tongued approach. For studies published prior to 2014, the four existing reviews were used to identify relevant articles. As those reviews also used PRISMA guidelines and similar inclusion criteria, this constituted a process fulfilling PRISMA criteria. Regarding studies published since 2014, *PsycInfo, PSYNDEX, PsycArticles, MEDLINE, and SocINDEX* databases were searched using the EBSCO search interface including studies published up until August 2019. The search phrases used for every database were: anorexia nervosa OR bulimia nervosa OR eating disorder AND therapeutic alliance OR therapeutic relationship OR working alliance OR bond OR helping alliance. The guidelines outlined in the PRISMA statement were used. Two independent raters reviewed the abstracts of all studies identified in the database search as well as those included in the previous reviews applying the previously specified criteria, specified below. For positively screened articles as well as those where the abstract did not provide sufficient information, the full text was screened and a final decision about inclusion was made. For articles that were rated differently, a decision was made through academic discussion.

Inclusion criteria were:

1. Participants of any gender and age, with an AN or BN diagnosis according to DSM-III-R, DSM-IV, DSM-5 [38–40], or ICD-10 [41].
2. Present or past psychotherapy of any kind, except exclusively group treatment.
3. An established quantitative measure of therapeutic alliance, i.e., a measure that was not first developed for the study in question.
4. At least one clearly defined measure of treatment outcome:
  - A quantitative measure of eating-disorder symptomatology,
  - The measurement of drop-out,
  - Diagnostic outcome (remission).
5. The availability of a statistical analysis regarding the relationship of therapeutic alliance and treatment outcome.
6. Published either in English or German.

After the process of study selection, data were extracted by the first author using a form based on PRISMA criteria as well as the summarizing tables of the existing reviews published on the topic [19, 33, 34]. For analyses conducted secondary as part of another study (e.g., RCT), we also looked into the primary papers for information regarding measurements and methods. We evaluated the quality of the studies using the quality assessment tool (QAT) provided by the National Heart, Lung and Blood Institute for observational cohort studies and cross-sectional studies [42]. Beyond this QAT, we also asked if the relationship of TA and outcome was the primary aim or a secondary analysis and used the sample size for evaluation. Regarding the first part of our first research question, we looked into statistical analysis involving any kind of correlational information. For the second part of this question, we concentrated on forms of analyses that either examined a predictive effect (e.g., regression analyses) or looked into correlations between the variables over time. It is important to note that especially the latter do not allow causal interpretations of the findings.

## Results

### Included studies

**Fig. 1:**
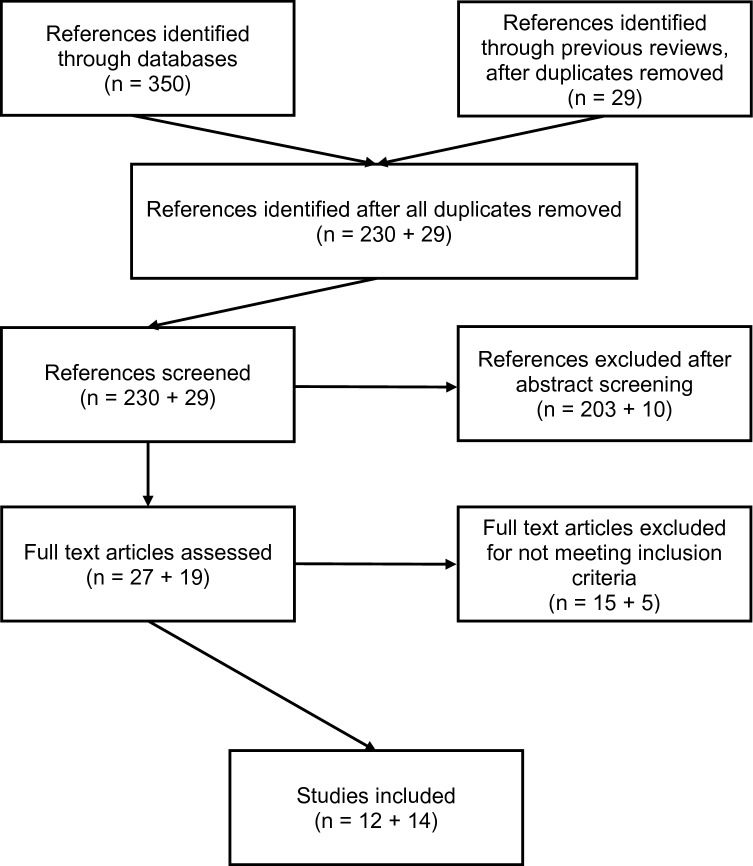
Flowchart of the screening process

The flowchart presented in Fig. 1 shows the process of study screening. The two independent raters agreed regarding the inclusion of 238 of the 259 articles. For the 21 articles where raters did not agree, full texts were screened and discussed until consensus was reached. Interrater reliability was 92%, with a Cohen’s kappa of 0.65 which can be rated as good [43].

26 articles were included. They consisted of 14 articles extracted from previous reviews and 12 articles that were found through our additional search. They can be put in the following categories: 13 articles examined patients with AN: five with adults, seven with adolescents and one with a mixed aged sample. For BN, nine articles were found: eight with adults and one with adolescents. Studies with a mixed sample (*n* = 4) will be reviewed in a separate paragraph. An overview of all included studies is given in Table 1. Citations of the included studies are tagged with an asterix in the bibliography.

**Table 1:** Overview of all included studies

Citation	*n*	Sample	Dx	Treatment	Setting	Gender/main ethnictiy	TA measure/point in time	ED measure	Quality assessment (NHLBI)	Outcome associated with TA
Accurso et al. [59]	80	Adults	BN	ICAT CBT-E	Outpatient	90% female	WAI-SR	Frequency of BPE	Good	Significant positive effect of TA on outcome (after controlling for previous outcome)
							Sessions 2, 8, 14 1 week FU			
										Significant positive effect of outcome on TA (after controlling for previous TA)
				21 sessions		88% white				
Bourion-Bedes et al. [52]	108	Adolescents	AN	IT SP	Inpatient and outpatient	94% female	HAQ-CP	Weight, EAT-26	Good-fair	TA significant predictor of reaching target weight
	85	Parents								
							Sessions 3, 6 and at reaching target weight			Significant relationship between TA and less time needed until reaching target weight
				No information about duration of treatment						
	23	Therapists								
Brown et al. [44]	65	Adults	AN	CBT	Outpatient	99% female	WAI-SR	Weight, EDE-Q, completion	Good-fair	No significant effect of TA on completion or BMI
				Sessions: mean = 31			Session 6, end of treatment			Weight gain significantly linked to later TA
						81% white				
Constan-tino et al. [60]	220	Adults	BN	CBT IPT	Outpatient	100% female	HAQ	EDE, PFF	Good-fair	Significant positive effect of TA on outcome (after controlling for previous outcome); some differences between therapies
				19 sessions						
						77% white	Session 4, 12			
										Significant positive effect of prior symptom change on later TA only in IPT
Forsberg et al [54]	38	Adolescents	AN	FBT	Outpatient	87% female	WAI-O	Weight, restraint-question from EDE	Fair	No significant effect of parents’ TA while controlling for early recovery
		Parents								
						76% white	Session 3, 4 or 5			
										No analyses regarding the influence of adolescents' TA on outcome
				24 sessions						
Forsberg et al. [53]	78	Adolescents	AN	FBT 24 × 60 min sessions	Outpatient	91% female	WAI-O	weight, restraint -question from EDE	good	Significant positive effect of TA on outcome measured as “partial remission”—no significant effect on outcome measured as “full remission”; after controlling for early change
							Session 3, 4 or 5			
						76% white				
				AFT 32 × 45 min sessions						
Hartmann et al. [66]	43	Adults	BN	PDT	Inpatient/day clinic	93% female	HAQ	EDI-2, SIAB	Good-fair	No significant effect of TA on remission status
				Mean duration: 3 months						
							Sessions 3 and every 8th session after			
Hughes et al. [55]	106	Adolescents	AN/EDNOS	FBT PFT	Outpatient	88% female	HRQ	Weight, EDE-I	Fair	Different results for FBT and PFT: FBT: significant difference between groups regarding fathers' TA (higher for early responders)
		Parents		18 sessions			Session 3			
										PFT: significant difference between groups regarding patients' TA (higher for early responders)
Isserlin and Couturier [56]	14	Adolescents	AN	FBT	Outpatient	100% female	SOFTA	Weight, EDE	Fair	Significant positive effect of parents' early TA on weight and patients' early TA on EDE
		Parents		Sessions: *M* = 13 (range 4–26)			Session 1, 2, 3, mid-treatment (median: session 8) end of treatment (median: session 12)			Significant difference regarding drop-out dependent on parents' mid- and late-TA
Jordan et al. [45]	56	Adults	AN	SSCM IPT CBT	Outpatient	100% female	VTAS-R	Completion	Good-fair	Significant effect of TA on completion (higher TA, less drop-out)
							Session 1–5			
				20 sessions						
Loeb et al. [61]	81	Adults	BN	CBT IPT	Outpatient	100% female	VTAS-R	EDE—purge frequency	Good	No significant effect of TA over time on outcome
							Session 6, 12,18			Significant positive effect of early TA on outcome
				20 sessions						
										No significant effect of symptom change on later TA
Marzola et al. [51]	173	Mixed age *M*: 24.9 SD: 9.7	AN	PDT	Inpatient emergency admissions	100% female	WAI-SR	Weight, EDE-Q	Poor in regards to our question	Significant correlation between TA and BMI at discharge (after controlling for baseline BMI)
				Mean duration: 36 days		100% white	Discharge			
Pelizzer et al. [62]	62	Adults	BN/OSFED	CBT	Outpatient	92% female	WAI-SR	Weight, EDE-Q	Good-fair	No significant effect of early TA change on outcome (high TA at session 1)
						87% white	Session 1, 4, 10			
				10 sessions						
Pereira et al. [57]	42	Adolescents	AN	FBT	Outpatient	91% female	WAI-O	EDE-I, completion	Good-fair	Significant correlation between parents' TA and drop-out
		Parents								
				12 months (20 sessions) vs 6 months (10 sessions)		74% white	Session 6, 16			Significant correlation between adolescents’ TA and early weight gain
										No significant effect of TA on outcome (EDE)
Raykos et al. [63]	112	Adults (> 16 years)	BN	CBT-E	Outpatient	99% female	HAQ-II	EDE-Q, completion	Good-fair	Significant correlation of TA and outcome
										No significant predictive effect of TA on completion or outcome
				20 sessions			Session 2, 10, 20			
Rienecke et al. [58]	56	adolescents (age *M* = 15,8; range: 12–24) parents	AN	FBT	Day program	93% female	WAI (regarding the whole treatment team)	Weight, EDE-Q, completion	Fair	Significant predictive effect of patients' TA on EDE-Q at discharge
				Mean duration: 27.6 days						
										No significant effect of TA on completion or weight
						93% white				
							Session 2, discharge			no significant effect of parents' TA on outcome
Sly et al. [46]	90	Adults	AN	Based on NICE guidelines no further information	Inpatient	97% female	WAI-S	Completion	Good	Significant predictive effect of early TA on completion
						98% white	Week 1, week 4, discharge			
										No significant effect of early TA change on completion
Sly et al. [47]	90	Adults	AN	Based on NICE guidelines, no further information	Inpatient	97% female	WAI-S	Staff-initiated premature discharge vs. patient-initiated premature discharge	Good	No significant effect of TA on reason for drop-out
						98% white	Week 1, week 4, discharge			
Stiles-Shields et al. [48]	63	Adults	SE-AN	CBT-AN SSCM	Outpatient	100% female	HRQ	Weight, EDE-I	good-fair	Significant predictive effect of early TA on EDE-I subscales at 12-month FU
							Session 2, 15, 30			
										No significant effect of early TA on other outcomes
				30 sessions						Significant predictive effect of late-TA on weight, EDE global and EDE subscales at discharge and 12-month FU
Treasure et al. [64]	125	Adults	BN	CBT ME	Outpatient	100% female	WAI	Frequency of BPE	Poor in regards to our question	Significant correlation between therapists' TA subscale "task" and outcome
							Session 4			
		Therapists								Significant correlation between patients' TA subscales “Task” and “goal” with outcome
				Study on first four weeks of treatment						
Turner et al. [69]	94	Adults	Mixed	CBT	Outpatient	95% female	WAI-SR	EDE-Q (early change)	Good-fair	Marginal significant predictive effect of TA on later change in symptoms
				20 sessions (min. 10 sessions, max. 40 sessions)						Significant predictive effect of early change in symptoms on TA
							session 6			
van der Kaap-Deeder et al. [72]	53	Mixed age *M* = 21.1 SD = 5.5; range = 14.6–44.3	Mixed	Specialized ED treatment, no information on duration	Inpatient	100% female	WAI-SR subscale bond	EDI-II subscales: drive for thinness, bulimia, body dissatisfaction	Fair	Significant predictive effect of TA on body dissatisfaction (1 year after start of treatment)
							After 3 months of treatment			
Waller et al. [71]	93	Adults	Mixed	CBT-ED	Outpatient	97% female	WAI-SR	EDE-Q, completion	Good	Significant predictive effect of early change in TA on overall change in EDE-Q
				10 sessions						
							Session 1, 4, 10 3 months FU			
										No significant predictive effect of TA on completion
Waller et al. [70]	44	Adults	Mixed	CBT	Outpatient	96% female	WAI-SR	Weight, EDE-Q	Poor in regards to our question	No significant correlation between TA and outcome
				Mean sessions: AN: 34 BN: 19						
							Session 6			
Wilson et al. [65]	120	Adults	BN	CBT ST (with additional medication or placebo)	Outpatient	100% female	HRQ	Number of daily BPE, remission, completion	Good-fair	Significant effect of mean TA on remission status
							Session 5, 12, 20			
										Significant effect of prior symptom change on TA at session 12
										No significant effect of TA on subsequent symptom change
				20 sessions						
Zaitsoff et al. [68]	80	Adolescents	BN	FBT SIP	Outpatient	98% female	HRQ	EDE objective binge/purge episodes	Poor in regards to our question	Significant correlation between mid-TA (not late-TA) and outcome in SPT group
				20 sessions		64% white	Session 10, 20			
										No significant correlation between mid- and late-TA and outcome in FBT group

Not all studies included data on ethnicity, and hence, only for some studies percentages of main ethnicity can be described. Percentages are rounded to integers. *AN* anorexia nervosa, *BN* bulimia nervosa, *Dx* diagnosis, *ED* eating disorder, *AFT* adolescent focused therapy, *CBT* cognitive behavioral therapy, *CBT-E/CBT-ED/CBT-AN* cognitive behavioral therapy, specialized for eating disorders, *FBT* family-based therapy, *ICAT* integrative cognitive-affective therapy, *IPT* interpersonal therapy, *IT* individual therapy, *ME* motivation enhancement, *NHLBI* National Heart, Lung and Blood Institute, *PDT* psychodynamic therapy, *PFT* parent focused therapy, *SSCM* specialist supportive clinical management, *SIP* supportive individual therapy, *ST* supportive therapy, *TA* therapeutic alliance, *HAQ/HRQ* Helping Alliance/Relationship Questionnaire, *HAQ-CP* Helping Alliance Questionnaire for Children, Parents and Therapists, *SOFTA* System for Observing Family Alliances, *V-TAS* Vanderbilt Therapeutic Alliance Scale-revised, *WAI* Working Alliance Inventory (*s* short version, *r* revised version, *o* observer version), *BPE* binge-purging episode, *EAT-26* Eating Attitudes Test-26, *EDE* Eating Disorder Examination (*I* interview, *Q* questionnaire), *EDI-II* Eating Disorder Inventory II, *PFF* Purge Frequency Form; *SIAB* Structured Inventory of Anorexic and Bulimic Syndromes

### Anorexia nervosa: adults

Five articles analyzing four samples looked into the influence of TA on treatment outcome of adult patients suffering from AN [44–48]. Three of those samples received outpatient treatment, and one study took place in an inpatient setting. Three out of four studies showed significant predictive effects of TA on outcome.

#### Are therapeutic alliance and treatment outcome related and does therapeutic alliance positively predict treatment outcome?

Regarding the association of TA and outcome, all studies found significant relationships between these two variables. With regards to the predictive effect of TA on outcome, results are more heterogeneous. Brown and colleagues [44] did not find a predictive value of TA for treatment completion nor for weight gain. Inversely, in their correlational analyses examining the sequential relationships between weight gain and TA, they showed that weight gain (between session six and the end of treatment) predicted TA at the end of treatment (EoT). This effect remained significant after controlling for early TA. In contrast, Sly et al. [46] found a significant prediction of premature treatment termination (PTT) by TA at admission, with an odds ratio of 1.053 (95% CI 1.011–1.098), while other factors like illness duration, body mass index (BMI), and Eating Disorder Examination—Questionnaire (EDE-Q [49]) at admission, motivation, and coercion did not predict PTT. As the change in TA during the first 4 weeks did not influence PTT, they argued that the first impression of the TA seems to be an important factor for remaining in therapy. In their secondary analysis [47] comparing patients that initiated their PTT themselves versus those with a staff-initiated PTT, they found no group differences regarding TA. Similarly, Jordan et al. [45] found that TA had a predictive value for the completion of treatment. The group difference between completers and non-completers regarding TA was significant with a medium effect of Cohen’s *d* = 0.44. The results of Stiles‐Shields et al. [48] correspond with this positive effect of TA on outcome. They analyzed a sample of outpatients with severe and enduring AN, using the Eating Disorder Examination Interview (EDE [50]). They showed that early TA did not predict later TA and that early TA only predicted two subscales of the EDE (restraint and shape concern; 6–7% explained variance), but neither BMI nor EDE global at EoT or the 12 month follow-up. However, late-TA predicted BMI and EDE global at both EoT and the follow-up, after controlling for baseline BMI and EDE, respectively. The explained variance was between 7 and 10%.

#### Which additional variables influence the relationship between therapeutic alliance and treatment outcome?

None of these studies conducted further analyses into other variables that might influence the relationship between TA and outcome.

### Anorexia nervosa: mixed-age sample

Marzola and colleagues [51], also examining inpatients with AN (age *M* = 24.9, SD = 9.7), measured TA with the primary case manager only at discharge. Concerning the link between TA and outcome, they found significant correlations between improvements in both BMI and quality of life and TA at discharge, even after controlling for baseline BMI, quality of life, and duration of illness. As they only used correlative analyses, no conclusion can be drawn with regards to predictive effects.

Regarding other variables influencing TA, they found no differences in TA between groups with different AN subtypes or different durations of illness. Instead, state‐anxiety, depression, and motivational stage at admission were significantly associated with TA upon discharge. The motivation to change remained a significant predictor even after controlling for the other variables, hence posing a possible moderating variable. Unfortunately, no moderator/mediator analyses were conducted.

### Anorexia nervosa: adolescents

Seven articles analyzing six samples looked into the influence of TA on treatment outcome of adolescent patients suffering from AN [52–58]. Interestingly, none of those studies were conducted in a solely inpatient setting, with four samples of outpatients [53, 54, 56, 57], one sample of inpatients and outpatients [52], and 1 day clinic sample [58]. All studies showed predictive effects of TA on outcome.

#### Are therapeutic alliance and treatment outcome related and does therapeutic alliance positively predict treatment outcome?

Similar to the results of adult patients, all studies found a significant relationship between TA and outcome. In respect to the predictive value of TA on outcome, positive effects of TA on outcome could be seen in all samples. Nevertheless, there were various and partly quite specific correlations, dependent on the type of outcome as well as to who was asked about the TA—patients or parents.

In a randomized-controlled trial (RCT) comparing Family-Based Therapy (FBT) with Adolescent-Focused Therapy (AFT), Forsberg and colleagues [53, 54] split patients into groups by their remission status, defined by weight and EDE criteria. While they found no predictive effect of patients’ TA on full remission status, partial remission was significantly predicted by TA after controlling for early symptom change, with an odds ratio of 3.32. As partial remission was defined by reaching a pre-defined weight (> 85th percentile on CDC norms), this emphasizes the importance and impact of TA on weight restoration. Neither the difference between mothers’ and fathers’ alliance scores, nor the difference between adolescents’ and their parents’ scores predicted weight recovery. Parental alliance was not predictive of outcome after controlling for early recovery. Hughes et al. [55] focused on the role of early treatment response in their study comparing FBT with Parent-Focused Therapy (PFT), where the therapist only works with the parents, while the patient is weighed on a regular basis by a nurse but does not get direct therapeutic support. Interestingly in the PFT group, the parents’ TA did not differ between early responders and non-responders, but the adolescents’ TA to their nurse showed a significant positive effect of early response. In the FBT group, early responders had fathers with a higher TA. Bourion-Bedes et al. [52] looked into a mixed setting sample, with some of the adolescents being hospitalized and some being treated as outpatients in an FBT program. Adolescents with a higher TA (groups dichotomized by median split) showed to have a significantly shorter period of time until reaching their target weight. Furthermore, early patients’ TA was one of the strongest predictors for achieving the target weight, with a hazard ratio of 2.7, again showing how important the impact of TA is on weight restoration. Rienecke and colleagues [58] examined a sample of adolescents in an FBT day clinic setting, examining the correlation of TA with treatment completion as well as outcome. There was no significant TA difference between completers and non-completers. However, due to a relatively low attrition with only 8.9% non-completion, conclusions drawn from group comparisons are limited. Concerning outcome, parents’ TA was not predictive of treatment outcome, but patients’ early and late-TA showed a significant predictive effect for EDE-Q total score at EoT, with an explained variance of 20% (early) and 40% (late).

While those results emphasize the significant role of adolescents’ TA, with little impact of parents’ TA, some studies also found effects of parents’ TA. Isserlin and Couturier as well as Pereira et al. [56, 57] examined the relationship of TA with ED outcome as well as with attrition from treatment in their samples of FBT outpatients. They both found a significantly higher TA of the parents in the completer group, with no differences between patients’ TA in completers and non-completers. In their group analyses regarding remission status, Isserlin and Couturier [56] showed that a higher TA in parents was correlated with weight-based remission, while a better patients’ TA related to the EDE-based remission. Pereira and colleagues [57] though found no predictive effect of TA on EDE. Regarding weight, adolescents’ early TA correlated with early weight gain, while overall weight gain was higher in the group with higher late parental TA.

#### Which additional variables influence the relationship between therapeutic alliance and treatment outcome?

While no studies conducted moderator/mediator analyses, some variables influencing TA were reported. Forsberg et al. [53] found a higher observer-rated TA at session four in patients in the AFT group than in FBT, suggesting an influence of therapy type on TA. In the study of Pereira et al. [57], adolescents who had more severe symptoms on the EDE had lower TA scores. Accordingly, Rienecke et al. [58] showed that patients’ early TA at session two was negatively correlated to patients’ EDE-Q total score at baseline, but not to their weight, age, or duration of illness. Bourion-Bedes et al. [52] found that TA scores were lower for patients as well as parents and therapists in the inpatient setting—as those inpatients were more severely affected, this may also indicate that in cases of higher severity there is less TA.

### Bulimia nervosa: adults

Eight articles examining seven samples looked into the effect of TA on outcome for adults suffering from BN. Six of those samples were outpatients [59–65], and one study compared an inpatient setting to a day clinic [66]. Results were heterogeneous, with some studies finding no predictive effect, some studies finding small, but significant predictive effects.

#### Are therapeutic alliance and treatment outcome related and does therapeutic alliance positively predict treatment outcome?

The results for BN patients are more heterogeneous than for AN. Two studies [62, 66] conducting specific analyses on the predictive effect of TA on outcome did not find TA to be a significant predictor. However, they did not conduct correlational analyses, and hence, they cannot give an answer to the first part of our research question. In contrast, for the other five samples, a significant relationship between TA and outcome emerged.

Regarding the predictive value of TA for outcome, results varied between studies. Treasure et al. [67] found a significant correlation between early symptom reduction and patients’ score of TA at session four. The therapist’s rating of TA was also positively related to improvement, but only for the subscale “task agreement”. As they only analyzed the first 4 weeks of treatment, the temporal relationship between these variables could not be examined. Hence, the predictive effect of TA was not examined. Raykos et al. [63] showed significant correlations between mid-treatment TA and mid-symptoms, mid-TA, and post-symptoms as well as post-TA and post-symptoms, with a higher TA in those patients with less symptoms, even after controlling for baseline symptoms. However, in their regression analyses, they found no predictive value of TA on outcome. This led the authors to the assumption that early symptom change influenced TA positively, although they did not examine this particular effect statistically.

In contrast, Wilson et al. [65] found a small predictive value of mean TA (averaged over all measurement points) on remission status with a 2% explained variance in their RCT comparing different psychological and pharmaceutical treatments. Although this effect is quite small, in light of just 6% explained variance by treatment type and 4% by medication vs. placebo group, it is not negligible. Regarding the temporal pattern, in the overall sample, a significant effect of prior symptom change on later TA could be found. In the subsamples split by psychotherapy type, no significant effects emerged. However, in the subsample of active medication, early TA predicted subsequent symptom change and prior symptom change predicted TA at termination. In the placebo group, prior symptom change predicted TA mid-treatment. Similarly, Loeb and colleagues [61] found a small, but significant impact (2% explained variance) of TA (therapist factor) at session six on outcome at end of treatment. They additionally performed temporal pattern analyses separately for both treatment groups [Cognitive Behavioral Therapy (CBT) and interpersonal psychotherapy (IPT)]. Other than one particular effect of prior symptom change on the patients’ contribution to TA at mid-treatment in the IPT group, they could not find any temporal relationship between the frequency of purging and TA in either direction. Constantino et al. [60] analyzed the same sample, but a different instrument for TA. They found a predictive effect of early and mid-TA on post-treatment outcome in CBT (14% and 7% explained variance), while in IPT, only early TA reached significance (3% explained variance; *p *< 0.05). Accurso et al. [59] found significant predictive effects in both directions: higher previous TA predicted less symptoms, after controlling for prior symptoms and less symptoms predicted higher TA, after controlling for prior TA.

#### Which additional variables influence the relationship between therapeutic alliance and treatment outcome?

No studies conducted moderator/mediator analyses, but some variables influencing TA were reported. Treasure et al. [64] looked into the effect of treatment motivation, conceptualized by the “stage of changes” model. A small effect of the stage “action” at admission on later TA rated by patients emerged. This effect was not found for therapists’ TA ratings. Wilson and colleagues [65] looked into the treatment types [CBT vs. supportive psychotherapy (SPT)] and found no difference regarding the quality of TA. Raykos et al. [63] examined the relationship of TA with baseline psychopathology, i.e., the overall depression and anxiety level as well as interpersonal problems, but found no correlations. In contrast, Accurso et al. [59] found a negative correlation, with higher depression, anxiety, and emotional dysregulation leading to lower TA. Interestingly, this effect was only found in the overall sample and the ICAT group, while it did not exist for the CBT group. They did not find a therapist effect on TA. Regarding other baseline variables, specifically age, sex, BMI, eating-disorder severity, substance abuse history, and affective lability and their interactions with treatment, there were no significant predictors of alliance.

### Bulimia nervosa: adolescents

Only one study was found for the relationship between TA and outcome for adolescent patients with BN [68]. They conducted their RCT comparing FBT with Supportive Individual Therapy (SIT) in an outpatient setting. A significant correlation between mid-treatment TA (not late-treatment TA) and symptom reduction over the course of treatment was found only in the SIT group, while there was no significant correlation between mid- and late-TA and outcome in the FBT group. No analyses on predictive effects were reported. While no moderator/mediator analyses were used, some correlations between baseline variables and TA could be shown: a higher symptom severity was associated with less TA mid-treatment. Interestingly, this effect was significantly stronger in the FBT group than in the SIT group. A significant positive correlation between age and TA was found in the SIT group.

### Mixed samples

Four articles analyzing four samples looked into the influence of TA on treatment outcome of samples with mixed diagnoses. Three of those studies examined outpatient adults [69–71], and one study looked into a mixed-age sample (mean age = 21.1; SD = 5.5; range = 14.6–44.3) in an inpatient setting [72].

#### Are therapeutic alliance and treatment outcome related and does therapeutic alliance positively predict treatment outcome?

Waller et al. [70] did not find any significant correlation between TA and outcome. In contrast, the three other studies found a significant relationship. In regard to the predictive effect of TA, Turner et al. [69] found a statistical trend (*p *= 0.055) for an effect of TA at session six on subsequent symptom change, with 5.1% explained variance. Early symptom change predicted TA significantly with 9.6% explained variance. An analysis with TA as a possible mediator between early symptom change and later symptom change did not lead to a significant result. Van der Kaap-Deeder et al. [72] examined TA as a mediating variable in the relation between self-critical perfectionism and body dissatisfaction and found a significant predictive effect of TA on body dissatisfaction. Waller and colleagues [71] looked into a sample of non-underweight ED patients in an effectiveness study for a short 10-session CBT-E treatment. While TA did not have a significant effect on completion, early change in TA (between sessions one and four) did predict overall change in ED symptoms.

#### Which additional variables influence the relationship between therapeutic alliance and treatment outcome?

No moderator/mediator analyses were conducted on the relationship between TA and outcome.

### Evaluation of studies

To evaluate their explanatory power, we looked further into the quality of the studies included in this review. Only one of the studies used a power analysis to ascertain that their sample is large enough to find the effects examined. Only ten studies looked into TA as a primary aim, while 14 of the studies reported on secondary analyses of data primarily collected for other research questions. Five studies had a very small sample size (*n* < 50), fifteen studies had a sample size with 50 < *n* < 100, and only six studies used a sample size that was larger than 100. Seven studies used the 11-item version of HAQ that may be confounded because of questions relating to the previous benefit from therapy. Only four studies adjusted their analyses for possible confounding variables over and above the baseline values of the main variables of interest.

Those studies finding no correlation at all between TA and outcome [62, 66, 70] are in the minority. Hence, we have looked into some details that could explain those findings. Hartmann et al. [66] and Waller et al. [70] both examined a small sample with *n  *< 50, leading to the question if these studies are underpowered. Furthermore, Waller et al. [70] used a correlative design and had some flaws in the design, resulting in a QAT rating of poor in regards to our question. Pellizzer et al. [62] used exploratory analyses, looking into multiple different variables, which led to a Bonferroni correction and resulted in a significance level of *p *< 0.007. With a quite small sample size of 62 patients included in the analyses regarding TA, the power is probably quite low and possible effects may have not been detectable. Additionally, they only looked into early change of TA between sessions one and four and not TA itself. With quite high TA values at session one and hence limited possibility of change, these analyses might not have been best suited to investigate TA’s influence on outcome. While some other limiting factors, e.g., the use of the HAQ, late measurement of TA, explorative design or the mixing of diagnoses, also exist in other studies, it is noticeable that in these studies, they appear to be accumulated. Hence, the reliability and validity of the studies seems questionable.

Regarding the predictive effect of TA on outcome, we also compared the studies that come to different conclusions against each other. For adult AN patients, all studies had a sample size less than a hundred. Brown et al. [44] only measured TA rather late in treatment, at session six, not finding a predictive effect on outcome. Stiles-Shields and colleagues [73], finding a significant prediction, used the aforementioned HAQ. The other studies finding an effect [45–47] did not suffer from particular limitations other than the limited sample size, but with regards to outcome, they only looked into completion vs. non-completion. For adolescent AN patients, all studies found predictive effects of TA on outcome. While those studies also suffer from limiting factors as mentioned above, the factors vary between studies and there is no sign of a systematic bias.

For adult BN patients, Raykos et al. [63] conducted a theory-based, hypotheses-led study with a sample size > 100, the examination of TA as the primary goal and early measurement of TA—with the limitation of using the HAQ. Unfortunately, they present a theoretical explanation for not finding a predictive effect of TA without statistically examining it, hence missing out on the opportunity to strengthen the hypotheses, that early symptom change improves TA and not contrariwise. The studies supporting a predictive effect of TA again suffer from various limitations like small sample sizes, use of the HAQ and rather late measurement of TA. Still, there seems to be no systematic bias.

For the mixed samples, the study not finding any relationship [70] was discussed above and seems to be less reliable, with a small sample size of *n  *= 44, and the late measurement of TA at session six. The more recent study of Waller et al. [71] does not seem to suffer from particular limitations in finding no effect of early TA change on completion, but on general symptomatology. Turner and colleagues’ study [69], finding a marginally significant effect of TA on later symptom change, is limited by late measurement of TA. The study by van der Kaap-Deeder et al. [72] should be interpreted cautiously, as they only looked into a very specific outcome, using TA measured only after 3 months of treatment as a mediating variable in the relationship between self-criticism and body dissatisfaction. Additionally, they used a rather small sample (*n  *= 53), limiting the generalizability of their results.

## Discussion

The studies examined and summarized in this review draw a heterogeneous picture regarding the importance of TA for ED treatment outcome. The methods, i.e., the measurement of TA as well as the study design, and the statistical analyses varied widely between studies. Nevertheless, there are no indications of systematic bias, as limiting factors occurred throughout all studies, regardless of their results. Hence, some patterns emerge from the summary of those studies.

All studies of patients with AN found a significant relationship between TA and treatment outcome, regardless of the age structure or the setting of the sample. In contrast, two out of seven samples of adult patients with BN did not show any correlation. From the perspective of age, all studies conducted on adolescents found a correlation, while 3 out of 16 studies with an adult sample did not show a correlation.

With regards to the prediction of outcome by TA for adult AN patients, for three out of four samples, a predictive effect of TA on completion or outcome was shown, while one study found an effect of early change on TA but not the other way round. For adolescent AN patients, in all six samples predictive effects of TA on completion or outcome were found and came to the conclusion that patients’ own TA predicted a better treatment outcome. Three studies also found some, in parts quite idiosyncratic, effects of parents’ TA on outcome, while two studies did not find any effect of parental TA and one study did not look into this particular question.

For adult BN patients, in two out of seven samples, the correlative relationship between TA and outcome was not investigated, while the examined predictive effect did not emerge. Of the five studies finding a significant correlation, one study did not look into a predictive effect of TA and one study did not find a significant predictive effect of TA on outcome. Three studies showed small, but significant predictive effects (2% explained variance in two studies) of TA on outcome, with one of those also finding a significant prediction of early change on TA. The one study looking into adolescent BN patients did not perform analyses on the predictive effect of TA.

Regarding mixed samples, one of the four studies did not find any correlation, while the other three did show (marginally) significant predictive effects of TA on outcome, with one study also finding a significant prediction of early change on TA.

This summary suggests an answer to our third research question: Are there differential effects of TA on outcome, dependent on the sample characteristics? The diagnosis as well as the age of the sample examined lead to different results regarding the effect of TA on outcome. While TA seems to have a predictive effect for patients with AN, the effect for patients with BN remains unclear. Additionally, TA seems to be more important for adolescent patients. However, as the only study with adolescent BN patients did not look into the predictive effect, it cannot be determined if the effects found in the adolescent samples can be attributed to their age or their diagnosis.

None of the examined studies conducted a moderator or mediator analyses regarding the connection between TA and outcome. Due to that lack of data, we widened our research question to also look into variables influencing TA itself. As some studies did not look into this question at all, some did not find significant correlations with the examined variables, and some found significant correlations, it would exceed the scope of this review to give a detailed and balanced summary. However, with the intention of offering a perspective for further research, we want to mention the results of those variables that were examined in some studies. Significant correlations were found with: baseline anxiety, baseline depression, baseline motivational stage, therapy type (FBT vs. AFT), baseline symptom severity (EDE), higher emotional dysregulation, and age. The variables that were examined, but did not show a significant correlation included: AN subtypes, duration of illness, treatment types (CBT vs. SPT), baseline weight, baseline depression, baseline anxiety, baseline interpersonal problems, baseline eating-disorder severity, substance abuse history, baseline affective lability, therapist effect, age, and sex.

### Integration with previous research and suggestions for further research

The results of our study support the findings of the meta-analysis by Graves et al. and the systematic review by Zaitsoff et al. [33, 34], in showing that the effect of TA depends on sample characteristics, specifically ED diagnosis and age. Graves et al. [33] emphasized the importance of drop-out as a confounding variable, as they examined the influence of early symptom change on TA. Their results showed that influence is increasing in studies with more drop-out. In other words, patients who might not profit from early symptom change may instead be the ones who drop out of studies, leading to a potential bias in estimating the effect of early symptom change on TA. Supporting this hypothesis, our review found support for the predictive effect of TA on completion of treatment [45, 46, 57]. On the other hand, some studies failed to find a predictive effect of TA on completion [44, 58, 63, 71]. As those studies differ widely in regards to design, consideration of confounding variables, treatment setting, and patient groups, with evidence pointing in the direction of a larger effect of TA on completion for (adult) AN patients, it could be hypothesized that differential effects are emerging. There may be a subgroup of patients that needs a stronger TA before being able to open up to symptom change, and rather drop out of treatment if a stable TA is not achieved early. Hence, the establishment of a strong and stable TA would be a necessary precursor for treatment success for this group. The results of our review, with the effect being found especially in adult AN patients, match the clinical experience that adult AN patients, who are often chronically ill, show a high ambivalence to treatment and profit from a trustful TA.

In this regard, motivation to change may be a potential moderating factor for explaining those patterns. Highly motivated patients may interpret early change as a treatment success, leading to a higher TA. On the other hand, more ambivalent patients may be threatened by early symptom change and need a strong TA to remain in treatment and to be able to bear the anxiety-provoking symptom change. This effect of preventing drop-out may be less influential for younger patients, as they often do not have the possibility of dropping out of treatment with their parents deciding for them. That leads to fewer cases of drop-out [74] for adolescents than adults, hence less motivated patients still remain in treatment. TA could play an important role in this patient group to enhance the treatment compliance.

As the high ego-syntonicity and therefore ambivalence to recovery is a specific feature of AN [30], this could provide an explanation for the differences between AN and BN patients. BN patients are usually less ambivalent regarding giving up their symptoms, as this does not accord with their underlying wish of realizing control and thinness [75]. Hence, for two subscales of TA, agreement on goals and tasks, there may be less variance, with most BN patients agreeing with their therapists on tasks and goals. Second, the subscale bond may not be a necessary factor to accomplish change in more highly motivated patients.

While there is some research looking into the effect of motivation on TA for substance abuse disorders, most of it examines the effect of early motivation on TA, and not the other way round, with some results suggesting a small predictive effect of motivation on TA [76–78]. However, for other disorders as well as the effect of TA on motivation to change, the current state of research is barely existent.

As Graves et al. investigated the timeline of the bidirectional association between TA and outcome, we also wanted to look into this question. The first question relevant is the time point during which TA was measured for the first time. 13 out of the 26 studies measured TA during the first three sessions. However, five studies measured it during sessions four to five and eight studies measured it at session 6 or later. As the establishment of TA starts from the first meeting between therapist and patient, it seems recommendable to measure TA as early as possible, especially for the investigation of the relationship of TA and outcome over time. Two studies, examining adolescent AN patients, looked into the effect of TA on early change in symptoms and found a positive effect [55, 57]. Some studies also specifically looked into the positive effect of early change on subsequent TA, supporting the assumption of a bidirectional association over time. Four studies [59, 60, 65, 69] found positive effects in both ways, with TA improving outcome as well as symptom change improving subsequent TA. One study found a significant effect of symptom change on TA without an effect of TA on outcome [44], while one study showed the exact opposite effect (TA improving outcome, but symptom change not improving TA) [61]. Summarizing, there is strong evidence for a bidirectional, positive effect of TA and symptom change, hence suggesting that both factors can enter either a virtuous or a vicious circle. In regards to clinical treatment, these results implicate the importance of promoting both, a strong and stable TA and a focus on early symptom change to enhance the chances of a successful treatment.

Regarding the aforementioned differences in study designs, especially the assessment points, more frequently and periodically planned assessments could improve the insights into the bidirectional relationship between TA and outcome. However, a higher frequency of assessments comes with a higher strain on patients as well as clinical staff, therefore impeding the implementation of research in settings with high ecological validity (e.g., communal hospitals). Hence, brief and easy tools could provide an important instrument in improving the quality of research as well as the practicality of studies’ implementation. Duncan and colleagues [79] developed a four-item session rating scale, aiming to assess TA after every session for clinical use. While the shortness of the scale comes with some loss of validity and reliability, the opportunities provided by an instrument this easy to administer should lead to its consideration for “close-up” research designs. Additionally, brief instruments for assessing the therapists’ perception of TA could open up the opportunity to gain even more insight—for example into the effect of the synchronization of patients’ and therapists’ ratings regarding TA and its influence on the treatment success.

### Strengths and limitations

This study has some limitations. First, the included studies had large variations in their designs, limiting their comparability. Through our structured analysis, however, we related and compared them to each other, but this meant that different measurements of outcome had to be put on the same level. This led to a reduction of our analysis concerning detailed and differential effects. Second, while men are less likely to develop an ED, they still are underrepresented in the presented studies, with a third of the articles reporting on female-only samples and a quarter on samples of more than 95% women. As the ratio between men and women is estimated to be 1:10 in clinical samples and 1:4 in community samples [80], this does not sufficiently represent male patients. Third, the majority of the included studies examined samples of less than 100 patients, thereby reducing the statistical power. Moreover, no study included a power analysis before recruitment. Finally, due to the commonness of secondary analyses that often use correlative data analyses regarding variables at different points in time rather than more sophisticated methods like regression analyses, interpretation of those data must always be done carefully, bearing in mind that no causal conclusions are possible.

On the other hand, this study gives an up-to-date overview of the most current research on the topic of TA in patients with EDs. Twelve additional studies conducted, since the last review on the topic was included, extending the basis on which conclusions can be made. Additionally, this review adhered strictly to PRISMA guidelines, and gives valuable suggestions for further research.

## Conclusion

TA seems to have a predictive effect for patients with AN, especially regarding the completion of treatment for adults, and also regarding symptomatology for adolescents. The effect for patients with BN remains unclear. There seem to be a number of variables that may mediate/moderate this relationship, but the present state of research does not allow any specific conclusions in this respect.

To make studies comparable, the use of various measurement tools should be limited to those with a good theoretical basis and the decision for a particular instrument should be theory-based. To further enhance the understanding of the reciprocal relationship between TA and outcome, both factors should be assessed early and then on a regular basis throughout treatment. Sample sizes based on power-analyses as well as the report of effect sizes would enhance the quality of studies and facilitate a higher generalizability and comparability. To directly compare the different disorders, the examination and comparison of both AN and BN patients in the same setting might shed more light on the differential effects. Additionally, it would improve the explanatory power if the design of studies is theory-based, hypothesis-driven, and looks into TA as the primary aim, rather than through secondary, exploratory analyses. Studies should try to get more representative samples, which include different genders and ethnicities. As TA poses a variable with many possible connections to pretreatment patient characteristics as well as other process variables, the systematic examination of possible moderating or mediating factors should be conducted in future studies. Motivation to recover from the ED is a factor with the potential to explain differential effects between TA and outcome in different patient groups.

### What is already known on this subject?

There are indications of a relationship between TA and outcome. Previous reviews show heterogeneous results and only include studies up until 2014. The direction and specific nature of this relationship as well as possible influencing variables are still unclear.

### What this study adds?

This review includes studies up until 2019 concerning the question of the relationship between TA and outcome in eating disorders. It shows that TA seems to have a positive predictive effect on outcome and that this effect might depend on the composition of the sample, specifically age and diagnosis.

## Data Availability

Data sharing is not applicable to this article as no new data were created or analyzed in this study.
